# Using Artificial Intelligence to Advance Public Health

**DOI:** 10.3389/ijph.2023.1606716

**Published:** 2023-11-02

**Authors:** William B. Weeks, Brian Taliesin, Juan M. Lavista

**Affiliations:** ^1^ AI for Good Lab, Microsoft Corporation, Redmond, WA, United States; ^2^ PATH, Seattle, WA, United States

**Keywords:** artificial intelligence, public health, healthcare access, healthcare workforce, health equity

Artificial intelligence (AI) and its application to healthcare has captured the imagination of the media, particularly since the release of Generative Pre-trained Transformer (GPT) large language models in late 2022. In the medical literature, application of AI to medicine and clinical care has been more tempered, balanced, and long-lived, with exponential growth in the number of PubMed results over the past 20 years. AI has seemingly become integral to the future of healthcare, with the National Academy of Medicine embracing AI as part of a more agile future of medicine [1], the New England Journal of Medicine introducing a new series and journal dedicated to the topic [2], and the World Health Organization releasing guidance on its ethical use and governance [3].

Using AI to extend the provider workforce as an adjunct to clinical care by providing reasoned, coherent, and accurate information that supports diagnosis and treatment has been considered, although head-to-head comparisons of responses from physician experts and from AI models have been mixed. While GPT models can pass Medical College Admission Tests and National Medical Licensing Examinations [4] and provide answers to medical questions that are better received, longer, and more empathetic than those of physicians [5], results can be imperfect.

Humans, too, are imperfect. Over the last four decades, the Dartmouth Atlas Project [6] has documented marked unwarranted variation in health services utilization and care quality. That variation can lead to patient harm: in the United States, a substantial number of deaths attributed to diagnostic errors each year [7].

Because providers offer different levels of care quality in industrialized countries and there is no valid, publicly available way to reliably choose higher quality providers, it is not clear that provider-generated medical advice would be better than GPT-generated medical advice. Indeed, GPT-generated advice could be quite helpful to improve care quality for providers—supportively integrating the latest findings, care recommendations, and guideline adherence suggestions seamlessly into the clinical workflow.

The opportunity to improve care quality and access is perhaps greatest in places where access to care is restricted, raising the possibility that GPT could not only improve care quality for those who have access to healthcare, but also dramatically enhance access (and care quality) for the 4 billion people in the world who have limited access to medical care.

Importantly, there may be advantages of GPT that would accrue to those who currently have limited access to medical care. There is an estimated 17 years gap between scientific evidence and widespread application of evidence-based medicine to patient care, with the unwinding of harmful or non-beneficial care taking even longer [8]. Using GPT to integrate recent findings into care pathways could dramatically shorten that gap—allowing for almost real-time application of new evidence into clinical practice.

Those living in low- and middle-income countries (LMICs) might be the greatest beneficiaries of such use. While AI will only be effective if algorithms are safe, reliable, and representative of the populations being served, in LMICs, AI that is responsibly and ethically developed could be helpful in improving public health, in part because those living in LMICs often have broadband access and access to cell phones. Such connections would facilitate the use of GPT for populations to ask questions relevant to their health concerns. As an adjunct to publicly-funded community health workers—who might provide a boon to local economic conditions while upskilling the workforce—GPT could become integral to improving the effectiveness of LMIC healthcare systems: AI algorithms designed to improve diagnostic probability and accuracy might be used by such workers when specialist care is not immediately available. GPT and AI models could improve the efficiency of the healthcare workforce by directing only those who are likely to need specialized care to scarce and often distant care providers.

For example, approximately 450 million people have treatable diabetic retinopathy; however, there are only about 200,000 ophthalmologists, worldwide. There are not enough ophthalmologists available to diagnose the 450 million, much less treat them. However, use of AI-enhanced community-health-worker captured fundoscopic videos can first parse the video to identify high quality fundoscopic images (including providing immediate feedback on whether the captured images are of sufficient quality, or if the video needs to be recaptured), apply algorithms to provide risk estimates for diabetic retinopathy, and provide differential diagnoses including publication-informed next best steps. Only those with high risk for diabetic retinopathy would be referred to the specialist who—if not also responsible for the screening—can dedicate more time to treatment. The same model has been used for retinopathy of prematurity in Mexico, chronic otitis media in aboriginal children in Australia, and leprosy diagnosis in Brazil. This process—which could be widely used in LMICs—leverages available and relatively low-cost community health workers to capture images on phone that can be used to better use scarce, expensive resources, at scale.

Together, Microsoft and PATH are developing and applying AI models in LMICs across the care delivery spectrum. For instance, to address interpretation and data entry problems when using Rapid Diagnostic Tests (RDTs), we are developing a universal RDT reader that can use photographs of RDTs to interpret them, thereby reducing clerical errors, improving diagnostic accuracy, and helping governments more rapidly and accurately identify epidemic outbreaks. We have worked together to model the efficacy of meningitis vaccines, thereby helping governments more effectively and efficiently vaccinate their populations. We are working to improve logistics of supply distribution in remote areas of Africa.

But beyond improving patient care delivery, groups at Microsoft are addressing some of the social determinants of health that have global impact on health, such as sustainability, human rights, accessibility, and disinformation. While AI can improve the quality and efficiency of direct patient care, it can also be used to address the drivers of global health inequities.

Overall, we see great promise in the ethical application of responsible AI for the purposes of improving population health. Especially when applied to health and wellbeing, we need to ensure patient safety, co-design by and inclusiveness of those in LMICs, development of algorithms and outputs that are transparent, explainable, and built for equitable access with overall sustainability in mind. But the ethical use of AI in LMICs may allow those countries to leapfrog the often inefficient and sometimes antiquated or dangerous healthcare practices that have developed in high-income countries.

To be sure, AI and GPT models will continue to improve. However, early application might support efficient and effective medical decision making by local healthcare workers and national public health systems for populations in which the alternative is virtually no care, at all. Globally, we are suffering from a shortage of trained healthcare workers: building medical schools and training medical students and residents is a decades-long undertaking. As a more robust medical workforce is being developed, AI can accelerate health equity by becoming integral to public healthcare systems in both high- and low-income countries: they can dramatically improve care access, diagnostic accuracy, resource allocation efficiency, and workforce productivity.

## References

[1] Dzau VJ. Anticipating the Future of Health and Medicine-The National Academy of Medicine Prepares for Its Next 50 Years. JAMA (2023) 329(17):1445–6. 10.1001/jama.2023.4081 36939751

[2] Beam AL Drazen JM Kohane IS Leong TY Manrai AK Rubin EJ. Artificial Intelligence in Medicine. N Engl J Med (2023) 388(13):1220–1. 10.1056/NEJMe2206291 36988598

[3] World Health Organization. Ethics and Governance of Artificial Intelligence for Health: WHO Guidance. Licensce: CC BY-NC-SA 3.0 IGO. Geneva, Switzerland: World Health Organization (2021).

[4] Gilson A Safranek CW Huang T Socrates V Chi L Taylor RA How Does ChatGPT Perform on the United States Medical Licensing Examination? The Implications of Large Language Models for Medical Education and Knowledge Assessment. JMIR Med Educ (2023) 9:e45312. 10.2196/45312 36753318 PMC9947764

[5] Ayers JW Poliak A Dredze M Leas EC Zhu Z Kelley JB Comparing Physician and Artificial Intelligence Chatbot Responses to Patient Questions Posted to a Public Social Media Forum. JAMA Intern Med (2023) 183(6):589–96. 10.1001/jamainternmed.2023.1838 37115527 PMC10148230

[6] Dartmouth Atlas Project. Dartmouth Atlas of Health Care (2023). Available From: https://www.dartmouthatlas.org/ (Accessed October 5, 2023).

[7] Newman-Toker DE Nassery N Schaffer AC Yu-Moe CW Clemens GD Wang Z Burden of Serious Harms From Diagnostic Error in the USA. BMJ Qual Saf (2023) bmjqs-2021-014130. 10.1136/bmjqs-2021-014130 PMC10792094 37460118

[8] Institute of Medicine, Committee on Quality of Care in America. Crossing the Quality Chasm: A New Health System for the 21st Century. Washington, DC: National Academies Press (2001). 25057539

